# A Rare Case of Prostate Abscess With Pyelonephritis in an Adolescent Male

**DOI:** 10.7759/cureus.87345

**Published:** 2025-07-05

**Authors:** Hasan A Al-Ibraheem, George Hill

**Affiliations:** 1 Urology, Aneurin Bevan University Health Board, Newport, GBR

**Keywords:** abscess, adolescent, pediatric, prostate, pyelonephritis, transperineal drainage

## Abstract

Prostatic abscess is a rare urological condition in adolescents. We report the case of a 16-year-old male with no significant past medical history who presented with bilateral flank pain, dysuria, and systemic signs of infection. Imaging revealed bilateral pyelonephritis and a prostatic abscess. Initial conservative treatment with intravenous antibiotics alone showed no improvement, prompting ultrasound-guided transperineal aspiration of the abscess under general anesthesia along with antibiotics. The patient showed marked clinical improvement and was discharged. This case highlights the importance of including prostatic abscess in the differential diagnosis for adolescent males with persistent urinary symptoms and sepsis. It demonstrates the efficacy of transperineal drainage as a relatively safe and minimally invasive intervention in this population.

## Introduction

A prostatic abscess is an uncommon urological condition, typically seen in men in their fifth and sixth decades of life and very rarely encountered in the adolescent population [1]. The most frequently isolated pathogen in prostatic abscesses is *Escherichia coli*, followed by *Klebsiella pneumoniae*, *Pseudomonas aeruginosa*, *Staphylococcus aureus*, and occasionally other organisms [2]. The condition presents a diagnostic challenge in younger patients due to its rarity, overlap with more common pediatric infections, lack of routine prostate examination, and nonspecific symptoms. Treatment ranges from antibiotics to more invasive and surgical interventions [2]. We report a rare case of prostatic abscess associated with bilateral pyelonephritis in an adolescent male with no risk factors identified yet, apart from low BMI on admission.

## Case presentation

A 16-year-old male, previously fit and well apart from low body weight (47.4 kg, height 175 cm, BMI 15.5 kg/m²), presented with a three-day history of bilateral flank pain and dysuria. He had no significant past medical history and a normal developmental history. On examination, he was febrile and tachycardic with diffuse abdominal tenderness. He appeared sarcopenic, with a soft, non-distended abdomen. A digital rectal examination revealed a tender, enlarged, and boggy prostate. His blood tests showed a white blood cell count of 31.1 x 10^9/L (normal: 4.0-11.0 x 10^9/L), a C-reactive protein level of 221 mg/L (normal: <10), and a creatinine level of 157 μmol/L.

**Figure 1:**
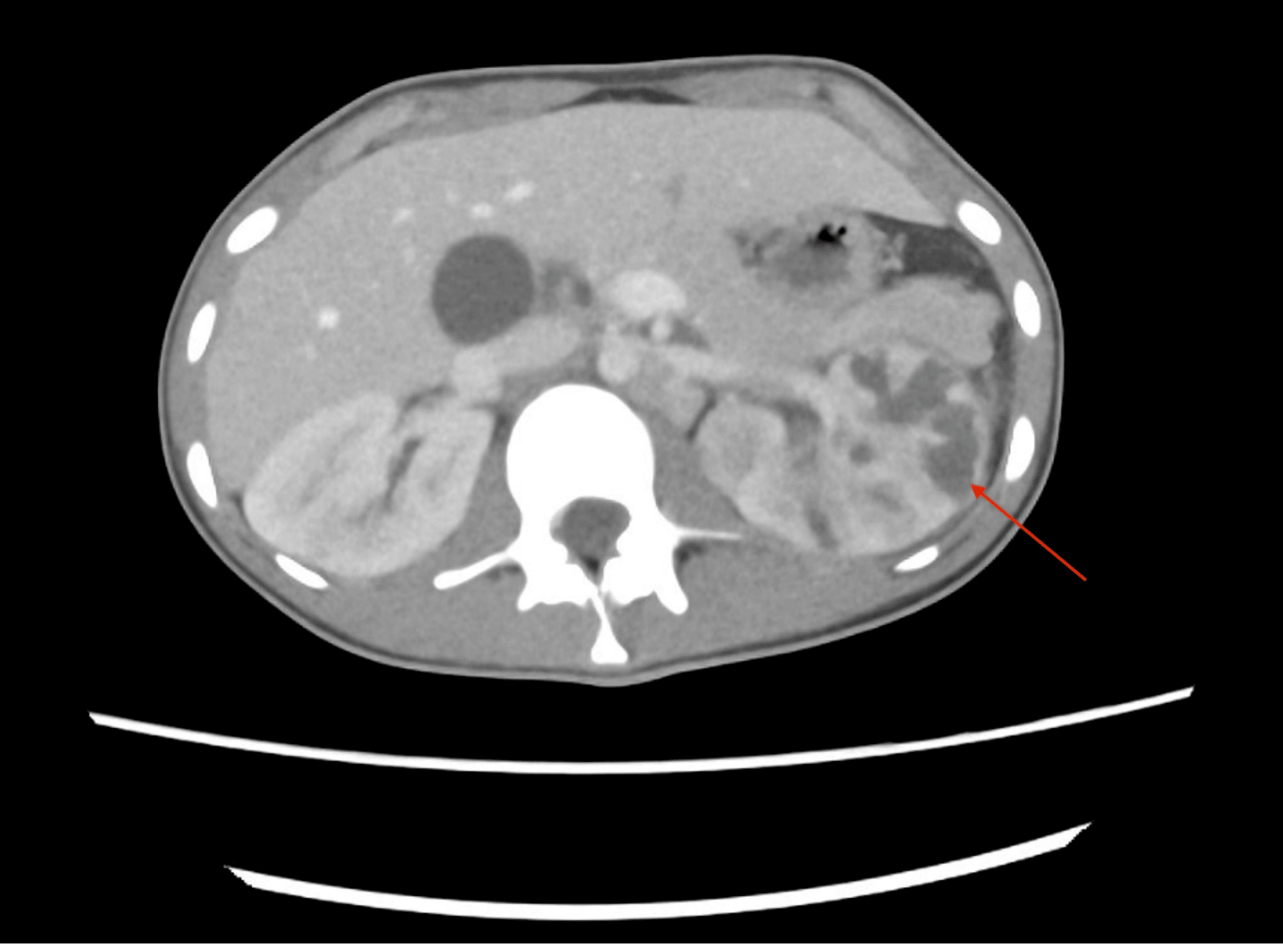
Contrast-enhanced scan of the abdomen in the axial view showing features suggestive of pyelonephritis CT: computed tomography

**Figure 2:**
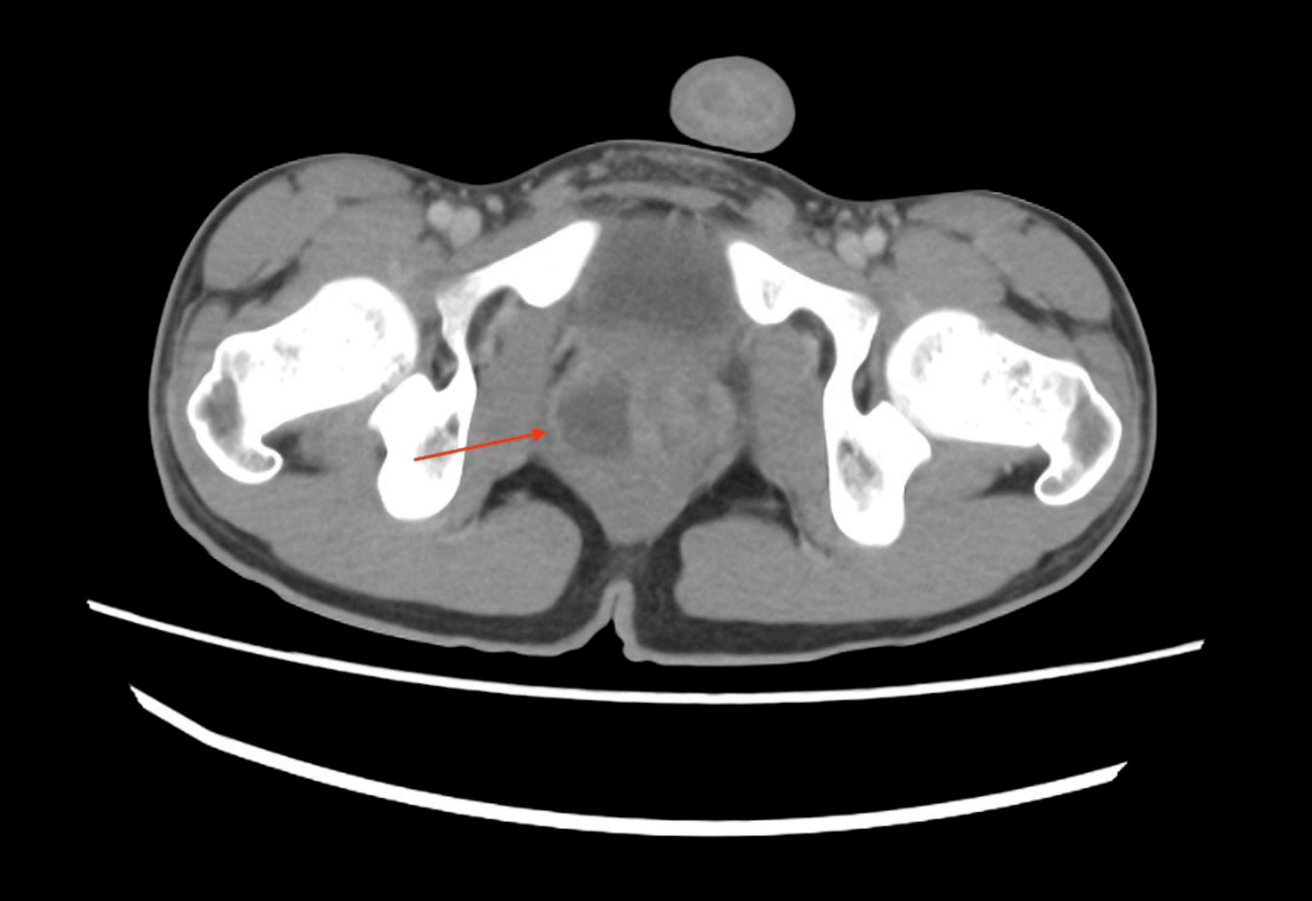
Contrast-enhanced CT scan of the pelvis in the axial view showing hypodense lesion in the right lobe of the prostate suggestive of prostate abscess CT: computed tomography

A contrast-enhanced CT scan of the abdomen and pelvis was performed. It demonstrated a grossly abnormal left kidney with multifocal areas of loss of corticomedullary differentiation, some of which were wedge-shaped in configuration. There was diffuse urothelial thickening of the left renal pelvis and proximal ureter. These findings were concerning for acute pyelonephritis with proximal ureteritis. The right kidney exhibited areas of early corticomedullary differentiation loss, consistent with early pyelonephritis (Figure 1). The prostate was enlarged, with a hypodense lesion in the right lobe, suggestive of a prostatic abscess (Figure 2).

The patient was managed empirically for sepsis; he was catheterized, blood and urine cultures were sent, and empirical intravenous antibiotics (piperacillin/tazobactam and metronidazole) were initiated following consultation with microbiology. Given the patient's young age, conservative management was initially preferred. However, due to the lack of clinical improvement, an ultrasound-guided transperineal aspiration of the abscess was performed under general anesthesia with a 14G needle to avoid transurethral or transrectal approaches in an attempt to reduce the risks of urinary incontinence or fistula. Approximately 3 mL of pus was aspirated to dryness and sent for microbiological analysis.

The culture results showed light growth of *Pseudomonas aeruginosa* and scant growth of *Enterococcus faecium*, while the urine culture revealed growth of *Pseudomonas aeruginosa*. Consequently, teicoplanin was administered for 14 days, and piperacillin/tazobactam was switched to oral ciprofloxacin 750 mg twice daily, based on the cultures' sensitivities and a discussion with the microbiology team. Nutritional support, including high-protein oral supplements, was initiated following the dietitian's assessment to address the patient's low BMI and poor oral intake during admission. The patient showed significant clinical and biochemical improvement. Inflammatory markers (white blood cells and C-reactive protein) trended downward, and the patient was discharged after 14 days to complete antibiotics in the community. His urethral catheter was successfully removed following discharge.

Further investigations were performed to identify potential underlying risk factors, including serological screening for hepatitis B virus, hepatitis C virus, and human immunodeficiency virus, which was negative; immunoglobulin levels were within normal range; complement levels were mildly reduced (C3 at 0.86 g/L (normal: 0.90-1.60 g/L) and C4 at 0.08 g/L (normal: 0.14-0.54 g/L)); and transthoracic echocardiogram revealed only trivial to mild pulmonary regurgitation and no vegetations.

He is awaiting an indirect MAG3 renogram, micturating cystourethrogram, and further assessment by the immunology team to address the mildly reduced complement levels. He has ongoing follow-ups with the dietitian.

## Discussion

A prostatic abscess is an uncommon but recognized complication of acute bacterial prostatitis, characterized by a localized collection of pus within the prostate. It predominantly affects men in their fifth and sixth decades of life and is rare in adolescents and children outside the neonatal period. Common risk factors include bladder outlet obstruction, recent urological instrumentation, prolonged catheterization, neurogenic bladder dysfunction, diabetes mellitus, and other chronic comorbidities [1].

*Pseudomonas aeruginosa* is one of the causative pathogens [2]. The clinical presentation can vary but typically includes lower urinary tract symptoms (dysuria, frequency, and urgency), acute urinary retention, fever, lower abdominal or back pain, and occasionally hematuria. On digital rectal examination, the prostate may be tender, enlarged, or fluctuant, raising suspicion of an abscess [3].

Diagnosis is confirmed radiologically. Transrectal ultrasound (TRUS) is a common initial imaging modality, detecting up to 80% of cases [4]. However, CT and MRI offer superior delineation of the abscess and assessment for extra-prostatic extension. CT is particularly useful in detecting renal and ureteric involvement [5], while MRI provides high-resolution soft tissue imaging [6]. In our case, we opted for a CT scan as it was more readily available in the emergency setting overnight, unlike ultrasound or MRI.

Initial treatment involves broad-spectrum antibiotics. While some cases respond to medical therapy alone, surgical drainage significantly reduces the duration of symptoms and hospital stays [2]. Minimally invasive techniques, including TRUS or transperineal aspiration, are preferred due to the reduced risk of complications. These can be performed under local or general anesthesia, and aspirated fluid should be sent for culture [7]. In refractory or multiloculated abscesses, transurethral deroofing may be necessary [8]. Open drainage is rarely indicated and reserved for extensive abscesses involving adjacent spaces such as the perirectal or perineal regions. Other complications of untreated or severe abscesses include fistula formation, sepsis, septic emboli, and even death [9].

Transperineal drainage of prostate abscesses may be less invasive, preserve ejaculatory function, and avoid urethral injury [10]. However, due to the rarity of the condition, there is insufficient data to support this approach definitively.

A literature review was conducted using the PubMed, Scopus, Embase, and EBSCOhost electronic databases, focusing on the following terms: “prostate abscess AND adolescence” and “prostate abscess AND paediatric”, with a focus on case reports. We excluded results in the neonatal period. We identified eight case reports, which are shown in Table 1.

**Table 1:** Summary of reported prostatic abscess cases in pediatric and adolescence age groups MRSA: methicillin-resistant *Staphylococcus aureus, *TRUS: transrectal ultrasound

Age	Pathogen	Possible risk factors	Treatment
5 [11]	Pseudomonas aeruginosa	Autism spectrum disorder, poor nutrition	Antibiotics, vitamins, and trace elements
14 [12]	Escherichia coli	Repetitive forceful influx of contaminated water into the urethra	Antibiotics only
15 [13]	MRSA	Previous history of MRSA infection	Transurethral unroofing of prostatic abscess and antibiotics
13 [14]	MRSA	No risk factors are known	Ultrasound-guided transrectal drainage and antibiotics
11 [15]	Methicillin-susceptible Staphylococcus aureus	Pulmonary hypertension	Ultrasound-guided percutaneous drainage and antibiotics
6 [16]	Staphylococcus aureus	Poststreptococcal glomerulonephritis	Surgical drainage (the approach is not clear) and antibiotics
15 [17]	Not specified	X-linked chronic granulomatous disease	TRUS-guided prostatic aspiration, transurethral resection and drainage of the prostate abscess, and antibiotics
12 [18]	Staphylococcus aureus	No risk factors are known	Transurethral incision of prostate abscess and antibiotics

Our patient is a 16-year-old with a reduced BMI who developed a prostate abscess, probably due to an ascending urine infection; hence, it was associated with severe pyelonephritis. After failing to respond to conservative management with intravenous antibiotics, we elected for transperineal drainage, which improved his condition. The patient will need further follow-up and monitoring to assess for any recurrence or complications.

## Conclusions

Prostatic abscesses, although rare in adolescents, should be part of the differential diagnosis in young patients presenting with persistent urinary and systemic symptoms unresponsive to antibiotics. Early imaging and intervention are crucial in preventing complications. Escalation from conservative to invasive management should be considered when there is no improvement or worsening of the clinical condition. In this case, transperineal drainage proved to be an effective method, resulting in a successful outcome. However, there is not enough evidence, and further studies are required about prostate abscess in this age group and its management. Follow-up and monitoring are crucial for young patients to detect any recurrence or late complications.
